# Controlling for performance capacity confounds in neuroimaging studies of conscious awareness

**DOI:** 10.1093/nc/niv008

**Published:** 2015-11-05

**Authors:** Jorge Morales, Jeffrey Chiang, Hakwan Lau

**Affiliations:** ^1^ Department of Philosophy, Columbia University, 708 Philosophy Hall Mail Code: 4971 1150 Amsterdam Avenue New York, NY 10027 USA; ^2^ Department of Psychology, UCLA, 1285 Franz Hall Box 951563 Los Angeles, CA 90095 USA; ^3^ Brain Research Institute, UCLA, 695 Charles E Young Dr. South Los Angeles, CA 90095 USA

**Keywords:** neural correlates of consciousness (NCC), signal detection theory (SDT), high threshold theory, EEG, unconscious perception

## Abstract

Studying the neural correlates of conscious awareness depends on a reliable comparison between activations associated with awareness and unawareness. One particularly difficult confound to remove is task performance capacity, i.e. the difference in performance between the conditions of interest. While ideally task performance capacity should be matched across different conditions, this is difficult to achieve experimentally. However, differences in performance could theoretically be corrected for mathematically. One such proposal is found in a recent paper by Lamy, Salti and Bar-Haim [Lamy D, Salti M, Bar-Haim Y. Neural correlates of subjective awareness and unconscious processing: an ERP study. *J Cognitive Neurosci* 2009,**21**:1435-46], who put forward a corrective method for an electroencephalography experiment. We argue that their analysis is essentially grounded in a version of High Threshold Theory, which has been shown to be inferior in general to Signal Detection Theory. We show through a series of computer simulations that their correction method only partially removes the influence of performance capacity, which can yield misleading results. We present a mathematical correction method based on Signal Detection Theory that is theoretically capable of removing performance capacity confounds. We discuss the limitations of mathematically correcting for performance capacity confounds in imaging studies and its impact for theories about consciousness.

## Performance confound in studies of consciousness

In the search of neural correlates of consciousness (NCC), subjects’ response to the presentation of a visual stimulus can be assessed by subjective or objective measures (Snodgrass and Shevrin, 2006; Seth *et al.*, 2008; Sandberg *et al.*, 2010; Irvine, 2013). Researchers who use subjective reports as measures of the state of awareness of subjects recognize the importance of controlling for confounding factors (Merikle *et al.*, 2001; Bachmann, 2009; Dehaene and Changeux, 2011; Sergent *et al.*, 2013; Li *et al.*, 2014; Bachmann, 2015). Ideally, when comparing a condition where subjects report consciously seeing a target against a control condition where subjects report not consciously seeing it, the difference between these two conditions should be conscious awareness only.

When looking for objective measures of conscious awareness, it is common that some researchers treat performance at chance level as a reliable indicator of unconscious processing (Eriksen, 1960; Dehaene *et al.*, 1998; Kouider and Dehaene, 2007). The inability to distinguish a stimulus from noise or from another stimulus, however, should not be immediately equated with lack of awareness. Performance, at any level, should rather be treated as a potential confound in consciousness research (Weiskrantz *et al.*, 1995; Lau and Passingham, 2006; Lau, 2008; Bachmann, 2009; Dehaene and Changeux, 2011; Aru *et al.*, 2012a; 2012b; Li *et al.*, 2014; Pitts *et al.*, 2014; Bachmann, 2015). In contrast, subjective reports are indeed a valid measure of conscious awareness. As such, we should isolate the influence of task performance capacity in any comparison between different levels of subjective reports of awareness. However, even among those persuaded by this logic, few actually conduct experiments to isolate performance capacity confounds. The main reason is, probably, that it is difficult to achieve it experimentally. Usually, when subjective reports of awareness differ, performance capacity also differs. This is true in most detection and discrimination tasks, as well as in paradigms like binocular rivalry, in which detecting changes in the suppressed image is harder (Wales and Fox, 1970).

Nevertheless, some attempts to control for performance capacity have been recently made in conscious awareness imaging studies. For example, Lau and Passingham (2006) conducted a study using metacontrast masking. By varying the stimulus onset asynchrony (SOAs) between stimulus presentation and mask presentation, they found two SOAs where performance capacity in a discrimination task was matched for each subject, and yet subjective reports of awareness differed. They reported specific hemodynamic activation in the prefrontal cortex in association with trials in the condition that generated the higher percentage of “aware” ratings. This study can be taken as a proof of concept that performance capacity confounds can be eliminated. However, the number of trials where subjects claimed consciously seeing the target differed only by about 10% between the two conditions. Admittedly, a problem with this approach is that it relies on a specific kind of stimulus: metacontrast masked shapes. For researchers interested in other perceptual paradigms, it is hard to see how this method of performance capacity matching could generalize.

Another study (Persaud *et al.*, 2011) matched performance between the normal sighted side of the visual field and the subjective blind side of the visual field in a hemianoptic patient, by presenting stimuli with low contrast to the patient’s normal visual field and high contrast to the damaged visual field to compensate for the defects in processing sensitivity. But this opportunity is specific to the availability of a single rare patient.

While these studies effectively eliminated the performance confound as such, other problems intimately interlinked when controlling for performance can still arise. For instance, when performance is matched by varying the stimulation conditions, as in Persaud *et al.* (2011), pre- and post-perceptual processing can obscure the interpretation of awareness-related activations (Bachmann, 2009). Another potential issue is that subjective reports can differ due to variations in how subjects are probed and not due to differences in performance or conscious awareness itself. Different scales (Sandberg *et al.*, 2010, 2011) or different criterion contents (i.e. different aspects of the experience subjects use for report) (Bachmann and Francis, 2014; Bachmann, 2015) can hinder contrastive analyses in imaging studies. Finally, another potential problem is that markers of specific conscious contents corresponding to the target stimulus have to be distinguished both conceptually and experimentally from the markers of conscious processes nonspecific to the target. When attempting to eliminate performance confounds, this distinction is relevant because nonspecific conscious processes can be shared by both correct and incorrect trials (Bachmann, 2015). Unfortunately, it would be complicated to control experimentally for all these potential confounds at once.

In an attempt to overcome these difficulties, Lamy *et al.* (2009) proposed a general method to control for the influence of performance capacity by comparing between subjectively conscious and unconscious conditions during an electroencephalography (EEG) experiment. Instead of trying to match performance experimentally, they proposed to correct for its influence mathematically, keeping stimuli at threshold constant across aware and unaware trials. In this article, we focus on this potentially promising method. We first expand on the logic of their methodology, trying to provide an intuitive explanation for the motivation behind it. Then, we show that the method and its assumptions are problematic from the perspective of Signal Detection Theory (SDT) and offer an alternative based on it.

Although we focus on Lamy and colleagues’ proposal, it is important to note that we do so because it is a useful case study that has general conceptual and empirical ramifications concerning an appropriate analysis of perceptual signal, performance capacity confound, and the neural correlates of consciousness. Thus, the concerns we raise regarding Lamy and colleagues’ correction method can be generalized to other neuroimaging studies and techniques, as well as to philosophical debates on consciousness and its relation to performance in general and to attention in particular (Block, 2007; 2010; Lau and Rosenthal, 2011; Prinz, 2012; Montemayor and Haladjian, 2015). Furthermore, other laboratories have already used their suggested method (Hesselmann *et al.*, 2011) and leading consciousness researchers like Stanislas Dehaene have recently praised them for having accomplished the “remarkable feat” of keeping both performance and stimuli the same and, thanks to “a perfect control,” having “confirmed [a neural] signature of conscious access” (2014, pp. 129–30). However, despite all the merits behind it, their correction method makes what we think are unsound assumptions about perception and consciousness. Hence, its limitations have to be considered when designing and analyzing imagining studies on the neural correlates of consciousness.

## Mathematical correction for performance confound: unconscious lucky answers

Lamy, Salti and Bar-Haim (2009) (LSB, henceforth) conducted an event-related potentials (ERPs) study on the neural correlates of conscious and unconscious visual processing where stimuli were constant across aware and unaware conditions. Subjects were presented with a 15 × 15 matrix of tilted lines (15°), some of which were slightly more tilted (25°) forming a 3 × 3 target square in one of four possible quadrants. A 15 × 15 matrix with tilted lines (25°) masked the targets after a short (∼25 to 100 ms, individually adjusted to achieve 25% conscious detection) or a long (∼37 to 112 ms, individually adjusted to achieve 50% conscious detection) exposure. Subjects made two judgments. First, a 4-alternative forced choice (4-AFC) regarding the quadrant where the target 3 × 3 square was presented. Then, a subjective judgment whether they were aware of the target or whether they were just guessing. Continuous EEG was recorded from 20 scalp regions during all trials and subjects’ responses were coded in the four following categories: subjects reported seeing the stimulus and correctly indicated its location (*aware-correct*), subjects reported seeing the stimulus and incorrectly indicated its location (*aware-incorrect*), subjects did not report seeing the stimulus and correctly indicated its location (*unaware-correct*), and subjects did not report seeing the stimulus and incorrectly indicated its location (*unaware-incorrect*). Note that in the last two categories subjects reported they were just guessing.

Confirming previous similar results (Sergent *et al.*, 2005; Del Cul *et al.*, 2007; Koivisto and Revonsuo, 2010; Batterink *et al.*, 2012), LSB reported a scalp-wide difference in the P3 waveform component (a positive voltage in the 300–650 latency range) in subjects’ ERPs between the *aware-correct* and *unaware-correct* conditions. They took this difference to reflect conscious processing. Critically, the comparison was focused on correct trials only (*aware-correct* vs. *unaware-correct*), as a direct comparison between all the *aware* and all the *unaware* trials would have involved a performance capacity confound. That is, awareness would have been confounded with overall performance since awareness co-occurred with higher performance rates. By comparing correct trials only, LSB matched performance in the sense that both conditions involve perfect accuracy, enabling thus a legitimate comparison between awareness and unawareness.

However, to really match performance between the conditions, LSB correctly realized the need to distinguish between two possible scenarios for trials in which subjects answered correctly and did not report seeing the target (the *unaware-correct* condition). It is possible that subjects unconsciously processed the visual stimulus, and therefore answered correctly. Alternatively, subjects could also have failed to process the stimulus, i.e. neither consciously nor unconsciously, and yet arrived at the correct answer by chance—in a 4-AFC task, random responding leads to an expected 25% chance of being correct. It is important to eliminate the influence of these correct-by-chance trials, because in comparing *aware-correct* and *unaware-correct*, the hope is not just to match performance as measured by sheer accuracy (in this case accuracy was 100% in both conditions). Rather, one would hope to match the underlying performance capacity. Only by removing the influence of the correct-by-chance trials in the *unaware-correct* condition one would be able to compare two conditions where the underlying performance capacities are matched (both at ceiling).

Thus, LSB developed a mathematical method to correct for the influence of those correct-by-chance trials (see Supplementary Material and LSB’s endnote 2). Their underlying idea is that by looking at the overall accuracy in *unaware* trials, one can estimate what percentage of trials in the *unaware-correct* category is correct by chance. In a 4-AFC task we would expect 25% of unaware trials to be correct simply due to chance.

In order to correct for this percentage of unaware-correct-by-chance trials, LSB further assumed that the ERPs for these trials should just look like the ERPs for *unaware-incorrect* trials. The intuition behind their logic is that both types of trial have in common that subjects’ brains failed to process the target. The only difference is that subjects were lucky in the correct-by-chance trials. With this assumption in mind, they attempted to subtract away the influence of the correct-by-chance trials on the set of unaware-correct trials. In summary, they assumed that the observed ERPs for overall *unaware-correct* is a weighted sum of the ERPs of the truly correct trials (processed-unaware-correct trials) and the ERPs of the correct-by-chance trials (unprocessed-unaware-correct trials). Thus, their correction method would get at the underlying ERPs for the processed-unaware-correct trials, which they call *unaware-correct chance-free* trials (see Supplementary Material and LSB’s endnote 2 for details).

After this correction, LSB still found significant differences in the P3 components of ERPs between the *aware-correct* and the *unaware-correct chance-free* conditions. Because now both conditions were supposed to include only truly correct trials where subjects processed the targets effectively, they argue that performance capacity was truly matched. Their logic is that their results now really reflect the signature of conscious processing, uncorrupted by confounds of performance capacity.

## Problematic assumptions of mathematical correction for correct trials by chance

LSB analysis implicitly incorporates some of the major assumptions behind what is often called in psychophysics a High Threshold Model (HTM) (Swets, 1961; Luce, 1963; Green and Swets, 1966; Macmillan and Creelman, 2005). In this section, we discuss a general HTM in the context of detection and discrimination, and its discrepancies with the more popular methods of SDT.

### High threshold models

**Figure 1:**
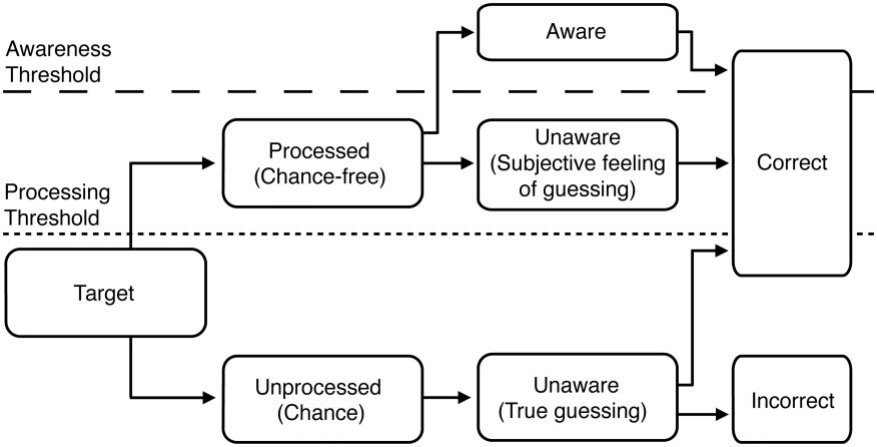
Schematic representation of LSB’s High Threshold Model conceptual framework. Unprocessed targets lead to some correct responses due to luck. When the signal crosses the processing threshold (dotted line), the target is processed and it will always lead to a correct response (because ‘processed’ in this context means ‘successfully processed’). Awareness requires crossing a further threshold (dashed line). We leave out from this schematic representation catch or lure trials (i.e. trials where no target was presented).

A key conceptual component of HTM is that there is a discrete boundary that separates two distinct conditions: effective processing, in which a target is being processed correctly, and ineffective processing, in which a target is not being processed at all (Fig. 1). According to HTM, mere background noise can never lead to true detection, which means that correct responses during unprocessed trials arise only from guessing. 

LSB seem to have in mind precisely this kind of model when discussing their experimental paradigm: “Because localization performance was clearly above chance, stimulus conditions were such that observers unconsciously perceived [i.e. processed] the target on average. Yet, on those individual trials in which the observers produced an incorrect response, *it is reasonable to claim that they did not perceive [i.e. processed] the target. Such trials were therefore defined as ‘no-perception’ trials*” (2009, p. 1442; emphasis added). Incorrect responses are a direct consequence, according to LSB, of a lack of processing of the target (bottom stream in Fig. 1) and, hence, of true guessing. LSB accept that perceptual processing is not sufficient for conscious awareness and, hence, that there can be processed unconscious targets (bottom half of top stream in Fig. 1). These trials are the ones that give rise to a subjective feeling of guessing. Note that in their framework the unaware processed trials are always correct (because incorrect trials are no-perception trials). Put simply, for LSB only targets (i.e. never pure noise) can cross the processing threshold. Conversely, if a target is not reported accurately it can be inferred that it was not perceptually processed. The distinction between processed and unprocessed stimuli is, then, sharp and clear.

Following this model, the only possible source of ambiguity is those unprocessed (and hence unaware) responses that are correct due to chance (upward arrow in bottom stream on Fig. 1). LSB suggest comparing *unaware-correct chance-free* and *aware-correct* trials to find the true neural correlates of consciousness. We conclude that the sharp distinctions between unaware-correct by chance, unaware-correct chance-free, and aware-correct trials that their proposal requires make sense only if something like HTM is assumed.

### SDT

**Figure 2:**
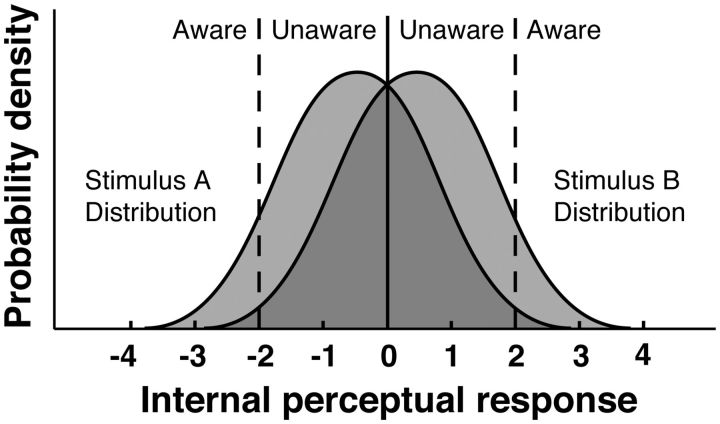
Signal Detection Theory model of perceptual awareness. The presentation of one of two possible stimuli evokes an internal response in the subject, falling into one of two Gaussian distributions. In each trial of a discrimination experiment subjects set a discrimination criterion (solid vertical line) and awareness criteria (dashed lines) against which they compare their internal response. Because the distributions overlap (darker area), it is possible (and quite common) that stimulus A is mistaken for stimulus B, or vice versa. Wrongly classified trials are reported as conscious if the internal response crosses the awareness criterion on the wrong side of the discrimination criterion.

Despite its *prima facie* intuitiveness, decades of psychophysics research have favored SDT over HTMs (Luce, 1963; Klein, 2001; Macmillan and Creelman, 2005). Rather than having binary “processed” and “unprocessed” internal states, according to SDT the presentation of a target gives rise in the subject to an internal perceptual response that lies on a continuum (Fig. 2). The strength of the internal response is hardly ever exactly at zero due to the presence of noise. In other words, a stimulus is hardly ever in an unprocessed state. The signal of a target is always corrupted by noise, and therefore, performance capacity is determined by the signal-to-noise ratio of the internal response. There is no magical point below which subjects always completely fail to process the target and above which they always process it successfully. 

According to SDT (Macmillan and Creelman, 2005), the presentation of a stimulus A or B in a discrimination task gives rise to an internal response in the subject (Fig. 2). The internal perceptual response varies from trial to trial, falling into one of two Gaussian distributions with equal variance and different means, depending on the stimulus presented and the subject’s internal state (i.e. noise). Subjects set a criterion against which they compare the internal response, which leads to the classification of the signal as being due to the presentation of stimulus A or B. The placement of the internal decision criterion can be determined by perceptual biases or by subjects’ response biases (Witt *et al.*, 2015). These can be influenced by preference, a strategy for maximizing the proportion of correct answers or expected value, subjective appearance (veridical or not) of the target, or attentional resources (Macmillan and Creelman, 2005; Rahnev *et al.*, 2011; Morales *et al.*, 2015). Because the distributions for the internal responses overlap, it is possible (and quite common) that stimulus A is mistaken for stimulus B, or vice versa. Additionally, trials are reported as *aware* when the internal perceptual response is strong enough to cross one of the outermost awareness criteria, and they are reported as *unaware* otherwise. Note that this allows for *aware-incorrect* trials when the internal response is drawn from the wrong distribution and yet it is strong enough to cross an awareness criterion (e.g. the right tail of the stimulus A distribution beyond the awareness criterion in Fig. 2).

Insofar as SDT rejects this strict dichotomy between perfectly processed and unprocessed stimuli, it is incompatible with HTM. But why prefer one model over the other?

### The argument from incorrect conscious trials

A specific problem of HTM regarding consciousness studies is that it cannot explain the presence of incorrect trials when subjects report being aware of a target. According to the model as conceived by LSB, if subjects are aware of a target, it has to be because it was successfully processed. Thus, the presence of *aware-incorrect* trials is a problem. LSB report a small, but not negligible, percentage of this kind of trials: 11% and 3.9% for short and long exposures, respectively. It is common practice in psychophysics to take into consideration lapse trials, i.e. trials where subjects did not witness the signal at all—sneezes or blinks are often blamed—or trials where nonperceptual problems, like motoric clumsiness, are accountable for the mistake. Lapse trials, however, are estimated at rates that go from 0% to 1% in the most lenient cases (Klein, 2001), which leaves LSB’s empirical results unexplained.

However, *aware-incorrect* trials are not uncommon and they can be seen in many other studies (Hesselmann *et al.*, 2011), and in some cases in high proportions (Lau and Passingham, 2006). Hence, the presence of *aware-incorrect* trials in LSB’s experiment is in conflict with the core assumptions behind their version of an HTM. In contrast, as can be noted in Fig. 2, *aware-incorrect* trials are an expected consequence of the SDT assumptions of our proposal. These trials are classified as aware and hence, despite being incorrect, should be accounted for when looking for the NCC.

### Empirical inadequacy of HTM receiving operating characteristic curves

**Figure 3:**
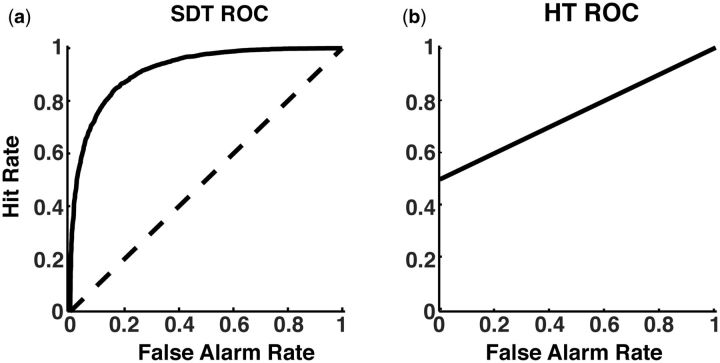
ROC curves comparison. **(a)** ROC curve as predicted by SDT. According to SDT, the trade-off between having more hits and false alarms when there is non-zero sensitivity is a non-linear relationship determined by the signal-to-noise ratio. A zero sensitivity scenario would yield a straight ROC line from zero to one (diagonal dashed line), where one can only increase hits by increasing the same amount of false alarms. A higher than zero signal-to-noise ratio means that the ROC curve will be curvilinear, where one can increase hits without increasing false alarms in the same proportion (solid curve) (i.e. performance above chance). ROC curve obtained from 10,000 simulated criteria for the same sensitivity level (*d′*=1). See Supplementary Material, including Matlab code, for details regarding the simulation. **(b)** ROC curve as predicted by HTM. According to HTM, the vertical intercept is determined by the proportion of trials where the subject successfully processes the stimulus. The trade-off between hits and false alarms follows a linear relationship.

What really convinced generations of psychophysicists that SDT is a superior model to HTM is the comparison of theoretical and empirical ROC (receiver operating characteristic) curves. An ROC curve is a plot of hit rate against false alarm rate. In a discrimination task (but the principle generalizes to yes/no, detection, and forced-choice tasks as well), a subject’s hit and false alarm rates produce one point on an ROC plot. By changing the subject’s criterion in different conditions to be more liberal (more hits and more false alarms) and then more conservative (less hits and less false alarms), multiple points on the ROC space can be plotted. According to SDT, when sensitivity is different form zero, an ROC curve should be curvilinear (Fig. 3a), whereas according to HTM the ROC should be a straight line (Fig. 3b). Most empirical ROC curves from human subjects in visual experiments typically look like the one predicted by the SDT model, and hardly ever look like the one predicted by HTM. This is a strong reason to prefer SDT models over HTM with respect to human visual perception (Krantz, 1969; Macmillan and Creelman, 2005), auditory perception (Green and Swets, 1966), and memory (Wixted, 2009; Dube and Rotello, 2012). 

We should note that in the memory literature, HTMs have enjoyed more popularity than in different perceptual modalities. In particular, mixed models (Aly and Yonelinas, 2012; Yonelinas and Jacoby, 2012), where recognition responses follow HTM and familiarity responses conform to SDT, have been well received, but they have also been criticized from the perspective of SDT (Wixted and Mickes, 2010). Here we are agnostic to this specific issue. We are not arguing that all HTMs are necessarily wrong. What we maintain here is that in the case of vision psychophysics, it is uncontroversial that SDT is much better supported by empirical data than HTM and that HTMs are inappropriate for conscious awareness studies. Their inadequacy lies on how they depict the internal representation of signal and noise, heavily underestimating the role of the latter. Analysis methods for vision that assume HTM rather than SDT are, thus, problematic. But how problematic is LSB’s HTM for conscious vision? How exactly might it have biased their results?

## A computer simulation to demonstrate the inadequacy of LSB’s correction method

We performed a computational simulation analysis to evaluate the degree of inadequacy of the correction method proposed by LSB. The idea behind it was to determine, assuming SDT is the correct model of perceptual processing (as the empirical evidence robustly suggests), how results of an idealized ERP experiment would look like using LSB’s correction method. As any other theoretical model of perception, SDT has explanatory limits. It is only within these limits that we attempt to assess the effectiveness of LSB’s correction method.

**Figure 4:**
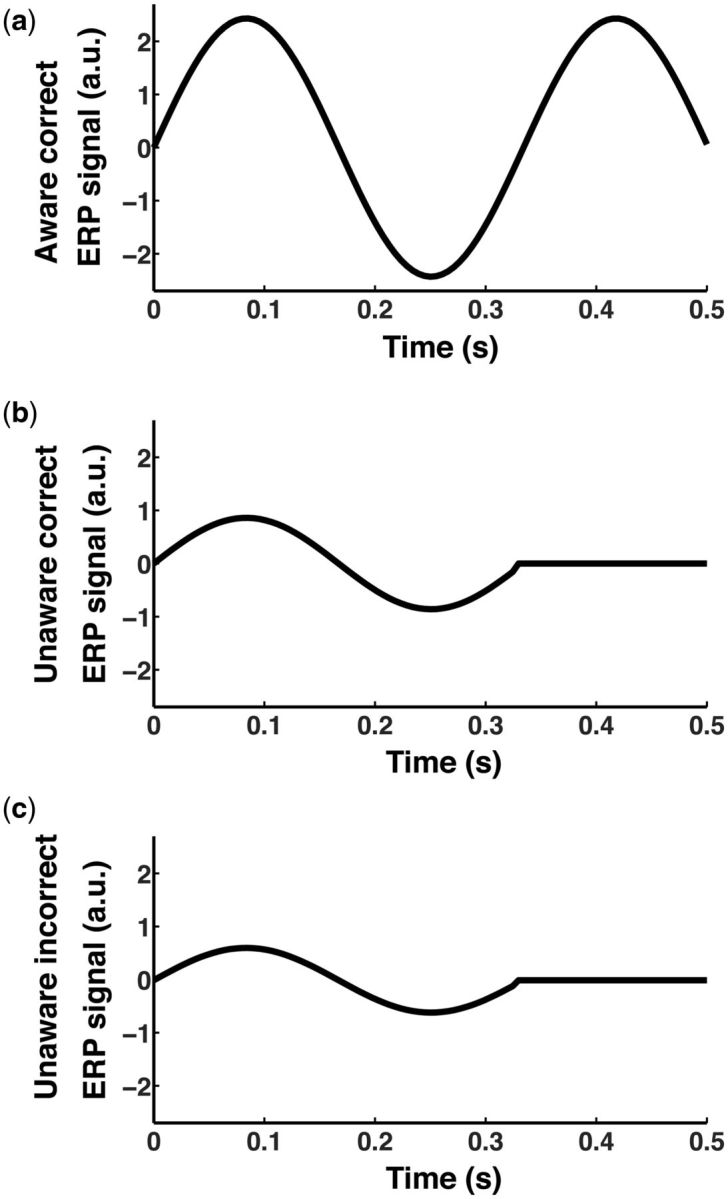
Average simulated waveforms under different conditions based on an SDT model. There is an extra third “bump” in **(a)**, the aware-correct trials, absent in **(b)** or **(c)**, the unaware trials. This late activity is meant to reflect activity that is specific to awareness. Activity intensity in **(c)**, the unaware-incorrect trials, is reduced compared to the higher activity in **(b)**, the unaware-correct trials. See Supplementary Material for details.

For simplicity, we assumed that subjects performed a two-choice discrimination task, which is analytically more tractable than a 4-AFC task and its results are trivially generalizable. The simulation consisted on distinguishing between two stimulus alternatives (A and B), and then reporting whether there was awareness of the target or not. It followed the SDT assumptions presented in section 3.2. The presentation of a stimulus along with noise is assumed to give rise to an internal perceptual response that varies from trial to trial and that falls into one of two Gaussian distributions depending on which stimulus was presented. Discrimination is made by comparing the internal response to a criterion. The trial is reported as aware if the strength of the internal response crosses one of the awareness criteria. For every trial, we made the strength of the internal perceptual response correlate with a hypothetical neural response and a corresponding ERP of an arbitrary electrode site. We modeled this ERP as a sinusoidal response over time, scaling the amplitude of the ERP response by the strength of the internal perceptual response sampled from either of the Gaussian distributions (Fig. 4; see Supplementary Material for technical details). 

For computational simplicity, we modeled perceptual processing as the ERP response from 0 ms to 333 ms. When the internal response was strong enough to cross the awareness criteria, the model assumes a constant brain signal is added to it, which may reflect a putative processing signature of awareness. For aware trials, then, we added an extra half cycle to the sinusoidal response so that there is a third “bump” in the ERP waveform (333–500 ms) (Fig. 4a). This extra cycle represents the differentiating processing uniquely associated with conscious awareness that is absent in trials without awareness (Fig. 4b and c). The idea is that by subtracting the *unaware* mean waveform from the *aware* mean waveform, if the unaware mean waveform is appropriately corrected for, we should be left just with activity properly related to awareness (i.e. the “third” bump). Despite its idealized nature, these simulations can help us determine the expected effectiveness of a performance correction method.

We note that neural responses associated with awareness need not arise late (>333 ms) and they need not be temporally dissociated from the purely classification processes. Finding the precise timing and localization of these signatures is the goal of imagining studies looking for the NCC. Hence, the simulations assumed the dissociated late timing for mere illustration purposes. The extra cycle associated with consciousness, then, could have been added earlier too (e.g. at ∼100 ms), as has been reported by different laboratories (Koivisto and Revonsuo, 2003; Pins and ffytche, 2003; Aru and Bachmann, 2009; Railo *et al.*, 2011; Andersen *et al.*, 2015; Rutiku *et al.*, 2015). Along with other simplifications (e.g. the use of a sinusoidal waveform or the fact that wavelength, symmetry and latency are constant with changes in internal response), these assumptions should not affect the main lesson to be drawn from this exercise. Its main purpose is to illustrate how a correction method that assumes HTM performs under reasonable SDT assumptions. To emphasize, we suggest this simple-minded model for ease of visualization and implementation only.

**Figure 5:**
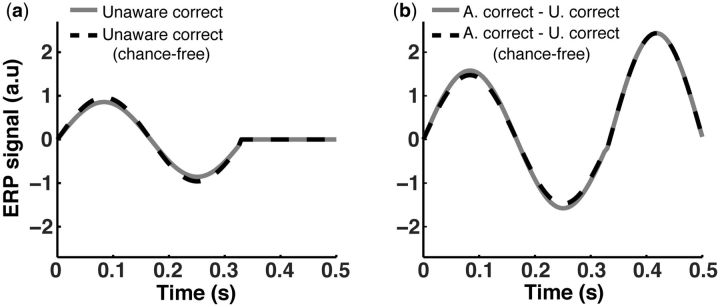
Simulated neural responses after LSB’s correction method. **(a)** Simulated unaware-correct (solid curve) and unaware-correct chance-free (dashed curve) activation using LSB’s (2009) suggested correction method. **(b)** The wave function result of subtracting the unaware-correct wave function (solid curve) and the unaware-correct chance-free wave function (dashed curve), respectively, from the awareness-correct wave function (Fig. 4a). It is evident from visual inspection that the influence of lucky responses was not sufficiently removed. Also, the activity during the early period was only marginally subtracted away with or without LSB’s correction method.

The results presented in Figures 4 and 5 were obtained after a 10,000-trial computer simulation (see Supplementary Material for technical details; the Matlab code used for generating all the simulations is provided as part of the Supplementary Materials). Figure 4 shows the ERP average responses under the different relevant conditions. In Fig. 5, we implemented the correction as described by Lamy *et al.* (2009; specifically, endnote 2). The *unaware-correct* response (Fig. 4b, and repeated for ease of comparison in Fig. 5a as the solid curve) is only marginally different from the *unaware-correct chance-free* response (Fig. 5a, dashed curve). This is the waveform obtained after applying the correction suggested by LSB’s method to eliminate performance confounds by lucky guesses. Hence, the influence of subtracting *unaware correct-by-chance* trials from *aware-correct* activations is only marginal. Both subtractive comparisons, namely, *aware-correct* minus *unaware-correct* (Fig. 5b, solid curve) and *aware-correct* minus *unaware-correct chance-free* (Fig. 5b, dashed curve), turn out to be almost the same, suggesting that the corrected unaware trials made a small contribution, if any, for singling out the signal specific to awareness. Concretely, in the latter comparison (Fig. 5b, dashed curve) there is still a clear residual activation during the first sinusoidal period of the ERP (0–333 ms), associated with the internal perceptual response strength in general, and not specifically to awareness, which occurs late in our simulations, i.e. from 333 ms to 500 ms (Fig. 4a). An optimal analysis where only the awareness signature response remains after a subtractive comparison should cancel out the early response, leaving just the late response that is specific to awareness. As it is clear from Fig. 5b, LSB’s method fails to single out the specific response associated with awareness when plausible SDT assumptions are in place, defeating the purpose for which it was originally devised. 

It is here that we can see the crucial, but flawed, role that LSB’s High Threshold assumption plays. They assume that the ERP response of *unaware-correct-by-chance* trials looks the same as the ERP response of *unaware-incorrect* trials (Fig. 4c): both are taken to be trials with unprocessed targets. Problematically, *unaware-incorrect* and *unaware-correct* do not look that different in the first place—the former’s amplitude is only about half smaller than the latter’s—so the *unaware-incorrect* waveform cannot have a very big influence on *unaware-correct* anyway. This is also observed in the actual ERP reported in LSB’s 2009 paper (their Fig. 3). It is of crucial importance to note that their results stayed basically the same regardless of whether they used *unaware-correct* or *unaware-correct chance-free* trials. In other words, their correction method affected in a negligible way their analyses, even though it was designed precisely to compensate for a significant underperformance during unawareness. This should be surprising for LSB since their assumed HTM implies that processed and unprocessed trials are radically different. Furthermore, in the P3 component during long-exposure trials (their Fig. 2) there is no difference between the amplitude of *unaware-correct* and *unaware-incorrect* trials. This is an important unpredicted fact in their theory that receives no comment. [We note that the difference between *unaware-correct* and *unaware-incorrect* was found to be significant in the P3 component in the parietal region in a follow up study (Salti *et al.*, 2012)].

On SDT, however, this type of outcome is to be expected because both *unaware-correct* and *unaware-incorrect* are trials that come from the inner partitions between the awareness criteria, where signal strength is weak (Fig. 2), and they are not necessarily very different in each of the two partitions. As a matter of fact, *unaware-incorrect* trials may even have higher internal response strength than *unaware-correct* trials (due to the overlap of the Gaussian distributions), making them in the end qualitatively similar. Thus, we conclude that LSB’s correction method only partially, and inadequately, removes the performance capacity confound.

### An SDT-based correction method

Having demonstrated the inadequacy of LSB’s correction method, we now show a way to perform a theoretically more adequate analysis based on SDT assumptions. The simulation presented in the previous section clearly established what the goal of such a correction should be, namely, to remove the ERP responses associated with mere processing in order to reveal the response that is specific to awareness and independent from performance. Like Lamy and colleagues, we are concerned with awareness as measured by subjective ratings (akin to confidence ratings as characterized within SDT). The distribution properties of the internal signal strength during a discrimination task are known when SDT is assumed, i.e. the internal perceptual response is drawn from one of two overlapping Gaussian distributions with equal variance and different means. Then, an appropriate correction for controlling for performance and factoring in any correct-by-chance trials is actually not difficult to achieve using standard SDT methods.

The primary assumption behind this correction is that activation intensity is linearly determined by the internal response. As it is clear from Fig. 2, an SDT model assumes that unaware trials have a lower mean internal response than aware trials. This fact can be used to correct for performance confounds between aware and unaware trials. The ratio of the mean internal response for aware and unaware trials is used as a scaling factor of the unaware mean waveform. By scaling up the weaker response in the unaware condition to approximately match the intensity of the stronger response in the aware condition, we can subtract away any activation due to magnitude difference in internal response (see Supplementary Material for technical details). Put simply, waveforms (but this is potentially generalizable to other types of imaging techniques like BOLD activity) of unaware trials during perceptual processing must be scaled up to match waveform amplitudes (or activation) of aware trials before they are subtracted from them.

**Figure 6:**
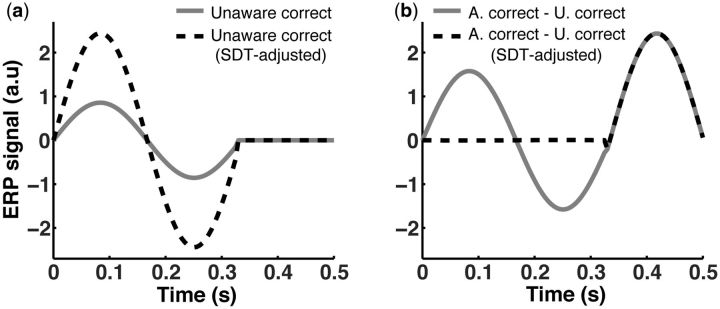
SDT-based correction method. **(a)** Simulated unaware-correct (solid curve) and unaware-correct SDT-adjusted (dashed curve) activations using our proposed SDT-based correction method (see Supplementary Material for details). **(b)** Aware-correct activation curve corrected by subtracting unaware-correct wave function (solid curve; identical to solid curve in Fig. 5b, repeated here for ease of comparison) and unaware-correct SDT-adjusted curve (dashed curve), respectively. When comparing the corrected aware-correct curve in this figure to the one in Fig. 5b (dashed curve), it can be easily noticed by visual inspection that the proposed SDT-based adjustment method robustly removes the task performance capacity confound during early processing stages, leaving just the awareness activation signature.

The correction from *unaware-correct* to *unaware-correct SDT-adjusted*, as we label it to distinguish it from LSB’s chance-free terminology, is presented in Fig. 6a (dashed curve). The subtraction of the scaled up unaware waveform should leave us mainly with the activations relevant to awareness (i.e. the third “bump”) in the simulated ERPs. Figure 6b shows the result of this process. For comparison, the subtraction *aware-correct* minus *unaware-correct* presented in Fig. 5b (solid curves) is repeated in Fig. 6b as well. Unlike LSB’s method, this adjustment method allows a significant difference between subtracting *unaware-correct* trials and *unaware-correct SDT-adjusted* trials. 

**Figure 7:**
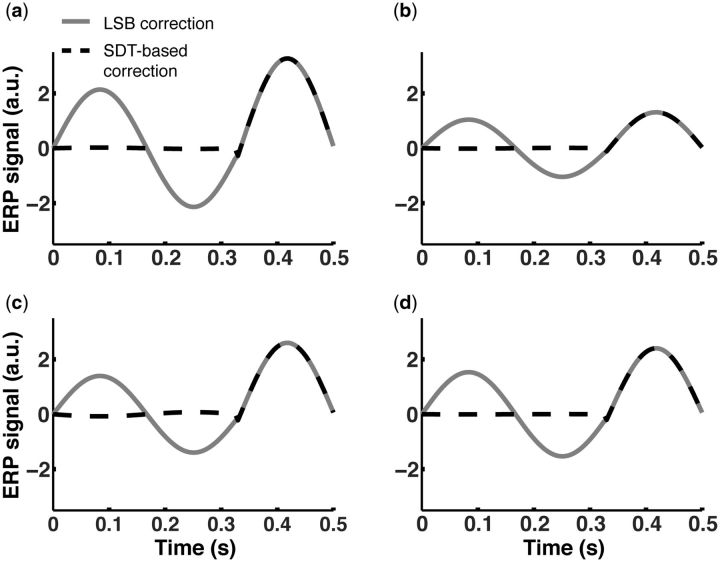
LSB’s and SDT-based correction methods stimulation results under different parametric assumptions. We ran 10,000-trial simulations changing the parameters for awareness criteria and sensitivity (*d′*). The solid curves represent the subtraction of unaware-correct chance-free from aware-correct activation (i.e. LSB’s method), and the dashed curves represent the subtraction of unaware-correct SDT-adjusted from aware-correct activation (i.e. our SDT-based correction method). **(a)** Sensitivity was kept constant and identical to previous simulations (*d′*=1). We assigned very conservative awareness criteria, i.e. the internal response strength had to cross ±3 on the x-axis of Fig. 2 for a trial to be classified as aware. **(b)** Sensitivity was as in (a), but we assigned very liberal awareness criteria (±0.5). **(c)** We held awareness criteria constant and identical to previous simulations (±2) and we assigned a higher sensitivity of *d′*=2. **(d)** Awareness criteria were as in (c), but sensitivity was set to a low level of *d′*=0.5. The results for all four variations look qualitatively the same to the simulation results presented in Figures 5–6. See Supplementary Material for details.

For the sake of completeness, we include in Fig. 7 results performing the same analysis with a different selection of parameters: better and worse performance (sensitivity *d’*) as well as more conservative and more liberal awareness criteria (see Fig. 7 caption and Supplementary Material for details on the parameters used). Even though there is a slight numerical variation, changing simulated sensitivity or awareness criteria left qualitatively intact the results thus far presented. The chance-free correction suggested by LSB is insufficient to isolate an awareness signature in the simulated ERP activation waveforms, while our SDT-based method is more robust to that end at the same time that it significantly reduces the worries regarding performance confound. 

## Discussion 

In order to discover the neural correlates of the exclusively subjective aspects of conscious awareness, eliminating performance capacity confound is a critical step. Lamy and colleagues’ effort should be commended for recognizing the importance of this issue, and for providing a novel and general method for dealing with this problem in a formal way. We recognize the intuitive appeal of its core logic as well as the importance and the potential impact that methods of its kind may have on the field. Unfortunately, whereas the overall concept behind the analysis is, *prima facie,* intuitive and appealing, it fails on a technical level due to its problematic assumptions.

The fact that the correction method proposed by LSB only minimally removes the performance capacity confound once plausible signal detection theoretic assumptions are made means that results based on it or on similar approaches have to be reassessed less optimistically. For instance, in their own study, LSB associated awareness with widespread activations. It would not be surprising that some of those activations are due to the failure to thoroughly remove the performance capacity confound. Other laboratories (e.g. Hesselmann *et al.*, 2011) have used LSB’s method trying to control for performance capacity and they found in an fMRI study that BOLD activity in the occipital and temporal areas was associated with awareness. But we know activity in some of these areas reflect internal response strength anyway (as they also predict task performance capacity), so their results may be merely due to the lack of complete removal of the influence of performance capacity. If this were the case, the view that these authors put forward, namely, that awareness may be associated with widely distributed activity in the whole brain, including visual areas, would be undermined. If an awareness signature response were correctly isolated, however, their findings may even turn out to be compatible with the view that awareness is associated with specific activity in a set of brain regions outside of the visual cortex, not directly involved in the generation of the internal perceptual response itself (Lau and Passingham, 2006; Lau and Rosenthal, 2011).

Our simulation results are not presented without misgivings. They are highly idealized and they make strong parametric assumptions regarding neural data. For instance, they assume that the internal perceptual response follows strictly Gaussian distributions and that the strength of the ERP (or whatever other neural response is analyzed, like BOLD activity) follows the exact same distributions. We know that SDT models are appropriate for human perceptual behavior because the underlying parametric assumptions have been validated by psychophysical measurements of ROC curves, which show that the Gaussian distribution assumption is empirically justified in most cases of visual perception. Nevertheless, when it comes to ERP data, relatively little is known about their statistical nature. If awareness modulates neural activity nonlinearly (Friston *et al.*, 1996), both the HTM and SDT corrections presented in this article would fail to reveal the corresponding neural correlates properly.

Another limitation of the present work, shared by LSB’s analyses, is that when contrasting unconscious and conscious activations, the latter could be revealing more than just the neural correlates of consciousness. These could also indicate brain activity present during conscious trials but unrelated to consciousness per se, like post-perceptual processing, working memory, or response preparation (Bachmann, 2009; Aru *et al.*, 2012b; Li *et al.*, 2014; Pitts *et al.*, 2014).

Finally, another limitation is that we assumed only one awareness criterion. This was done mainly for the sake of simplicity and computational tractability and it should not suggest that awareness is an on-off step function. Future work could pursue the effectiveness of this method with multiple criteria, which may more realistically capture the nature of subjective ratings. (Note that with enough criteria, the suggested type of modeling would, in practice, approximate a truly continuous scale.) Relatedly, it may be argued that there are subtle differences between confidence ratings (commonly used in SDT contexts) and awareness judgments (Overgaard and Sandberg, 2012). We acknowledge there are potential differences, but within the framework of SDT these two have been given similar treatments, in that they are both subjective ratings that can be modeled as responses separated by criteria.

With these caveats in mind, we think the conceptual ideas behind our SDT model are useful for the study of consciousness in both behavioral and imaging studies. Because this model is based on the localization of criteria along a decision axis, ratings of awareness can be dissociated from performance capacity, just as response bias can be dissociated from discrimination sensitivity (Ko and Lau, 2012; Maniscalco and Lau, 2012). Furthermore, for a single trial, given the internal response strength, the same stimulus could end up being classified as *aware* or *unaware* depending on where the criteria for awareness are placed. This is where HTM and SDT depart from each other more dramatically. Within SDT, for the same stimulus and the same internal response strength, the same subject could classify a trial as aware on one occasion and as unaware in a different occasion, depending on the localization of the subject’s awareness criterion. This boundary is determined by fixating a criterion that changes from subject to subject, from experiment to experiment, and most likely it even jitters from trial to trial.

Perhaps, the most important take-home message of the exercise of focusing on LSB is not methodological in nature. Rather, there is a broader conceptual point that we are hoping to advocate here. When controlling for performance capacity in imaging studies, researchers should focus on controlling for the internal response strength, and not just for adjusting the influence of mere flukes. In imaging studies of consciousness, this means isolating some kind of further processing which only happens during trials crossing the awareness criteria. Such is the logic behind our proposed correction method. Given the complexity of this problem as revealed by the limitations of our correction method described here, we believe that in order to address the issue of performance capacity as a confound, the best method so far is to create task conditions in which task performance is empirically matched, and yet reported subjective levels of awareness differ (Lau and Passingham, 2006; Rounis *et al.*, 2010). Though this may be difficult to achieve experimentally, we hope future research may be able to meet this important challenge.

## Supplementary Material

Supplementary Data

## Supplementary data

The Matlab code used to generate all the simulations is available as Supplementary Material is provided at *Neuroscience of Consciousness Journal* online.
