# Supplementary material for: Controlling for performance capacity confounds in neuroimaging studies of conscious awareness
Source: Neurosci Conscious. 2015 Nov 5;2015(1):niv008. doi: 10.1093/nc/niv008 (PMC5932880; doi:10.1093/nc/niv008)
Supplement: Supplementary Data [file suppl_data.zip › Supplementary Material.docx]

Supplementary Material

Here we describe the technical details of the 2-choice discrimination simulation reported in Figures 4-7. All simulations were made in Matlab (MathWorks, Natick, MA) and the code is provided as part of the Supplementary Material. We assumed equal Gaussian distributions for the internal response. The discrimination criterion (vertical solid line, Fig. 2) was simply the intersection of the two curves, which we arbitrarily placed at zero. The distributions for stimuli A and B were centered at *μ*=±*d’*/2, respectively, where sensitivity measure *d’* equals 1 in Figures 4-6, it equals 2 in Figure 7a-b and it equals 0.5 in Figure 7c-d. We arbitrarily set the awareness criteria at ±2 (vertical dashed lines, Fig. 2) for Figures 4-6 and to ±3 in Figure 7a-b and ±0.5 in Figure 7c-d. We stipulated the activation waveform to be a simple sine wave from 0 to 2π (Fig. 4a-c). For simplicity, we extended the domain to 3π, in which we added an additional wave to represent the extra activity in the *aware* condition (Fig. 4a). We scaled the domain to 500ms to maintain consistency with ERPs. The sine wave amplitude was directly proportional to the internal response. Specifically, the goal of the analysis was to recover the response that only appears from 2π to 3π (i.e. 333 ms to 500 ms), which was stipulated to be specific to awareness. Below, in Equation 1, *A_(unaware)_* and *A_(aware)_* are the amplitudes of the waveforms associated with unaware and aware trials at every time point, respectively. Finally, *x* represents the internal perceptual response, and *t* represents time in milliseconds.

$A_{(unaware)}=\left\{ \begin{aligned} x\sin t, 0<&t<333 \\ 0, t>333 \end{aligned} \right. A_{(aware)}=x\sin t$ Eq. (1)

We ran 10,000 trials per simulation (Fig. 4) and performed LSB’s correction method as presented in their endnote 2. We present the results of this correction in Figures 5 and 7, whereas we present the results of our SDT-based correction method in Figures 6 and 7. They derived the estimated waveform corresponding to unaware-correct chance-free trials as follows:

$A({UC}_{chance-free})=\frac{A\left( {UC}_{observed} \right)-\%{UC}_{chance}*A({UI}_{observed})}{1-\%{UC}_{chance}}$ Eq. (2)

where A is the amplitude of the waveform at each time point, UC_observed_ are the unconscious-correct trials, UI_observed_ are the unconscious incorrect trials, and %UC_chance_ is the expected percentage of correct trials by chance during the unaware condition such that:

${\%UC}_{chance}=\frac{\%Unaware Incorrect trials}{\% Expected Incorrect trials by chance}*\% Expected correct trials by chance$ Eq. (3)

Since we simulated, for computational simplicity, a 2-choice discrimination task rather than a 4-AFC task as was done in LSB, chance performance was 50% in all our simulations rather than 25%.

In out proposed correction method we can infer the awareness criteria using standard Signal Detection Theory (SDT) (Maniscalco and Lau, 2012). Note that the awareness criteria are slightly different from the standard discrimination criterion, but they are criteria all the same. So, in Equation 2 below we determine the awareness criterion *ac* by means of *C* and *I*, which represent the proportion of aware correct responses (analogous to hit rate) and the proportion of aware incorrect responses (analogous to false alarm rate), respectively. By using these rates instead of the standard hit rate versus false alarm rate calculation, we are able to determine awareness criteria (*ac*) in the same way that the discrimination criterion is determined. Thus,

$ac=-\frac{z\left( C \right) +z\left( I \right)}{2}$ Eq. (4)

where z(*C*) and z(*I*) are the z-scores of *C* and *I*.

Because we worked in standardized space (i.e. the standard deviation of the Gaussians is 1), knowing *ac* allows us to estimate the mean internal perceptual response for each partition (Fig. 2) using expected value:

$E\left( X \right)= \int x*p\left( x \right)=\frac{\int x*f(x)}{\int f(x)} dx$ Eq. (5)

This leaves us with the mean internal response for Stimulus A aware, Stimulus A unaware, Stimulus B aware, and Stimulus B unaware. Below, in Equation 6, 0 is the discrimination criterion, *ac*, as defined above, represents the awareness criterion, and *x* represents the internal perceptual response. Because the area under the curve of each partition is not 1, the means must be normalized. We illustrate the equations with Stimulus A as in Figure 2 (but it can trivially be altered for Stimulus B), and assumed *f(x)* to be a standard Gaussian distribution.

$M_{unaware}=\frac{\int_{0}^{ac} x*f\left( x | stimulus=A \right)dx}{\int_{0}^{ac} f(x|stimulus=A) dx}$ $M_{aware}= \frac{\int_{ac}^{-\infty} x*f(x|stimulus=A)dx}{\int_{ac}^{-\infty} f(x|stimulus=A)dx}$ Eq. (6)

We used the ratio of the means as a way to scale the waveform associated with the awareness condition and compare it to the unawareness condition to adjust the unaware waveform:

$A_{\left( unaware SDT-adjusted \right)}=\frac{M_{aware}}{M_{unaware}}*A_{(unaware)}$ Eq. (7)

Finally, we subtracted the A_(_*_unaware SDT-adjusted_*_)_ waveform from the A_(_*_aware_*_)_ waveform to obtain the distinctive awareness waveform thus eliminating the potential performance confound.
